# Novel internal regulators and candidate miRNAs within miR-379/miR-656 miRNA cluster can alter cellular phenotype of human glioblastoma

**DOI:** 10.1038/s41598-018-26000-8

**Published:** 2018-05-16

**Authors:** Subhashree Nayak, Meghali Aich, Anupam Kumar, Suman Sengupta, Prajakta Bajad, Parashar Dhapola, Deepanjan Paul, Kiran Narta, Suvendu Purkrait, Bharati Mehani, Ashish Suri, Debojyoti Chakraborty, Arijit Mukhopadhyay, Chitra Sarkar

**Affiliations:** 1 Department of Pathology, All India Institute of Medical Sciences, New Delhi, 110029 India; 2 Genomics and Molecular Medicine Unit, CSIR-Institute of Genomics and Integrative Biology, Mathura Road, New Delhi, 110020 India; 3 Department of Neurosurgery, All India Institute of Medical Sciences, New Delhi, 110029 India; 4 Proteomics and Structural Biology Unit, CSIR-Institute of Genomics and Integrative Biology, Mathura Road, New Delhi, 110020 India; 5 G. N. Ramachandran Knowledge Centre for Genome Informatics, CSIR-Institute of Genomics and Integrative Biology, New Delhi, 11020 India; 6 Academy of Scientific and Innovative Research (AcSIR), Delhi, India; 7 Department of Pathology, All India Institute of Medical Sciences, Bhubaneswar, 751019 Odisha India; 8 School of Environment and Life Sciences, University of Salford, Manchester, United Kingdom

## Abstract

Clustered miRNAs can affect functioning of downstream pathways due to possible coordinated function. We observed 78–88% of the miR-379/miR-656 cluster (C14MC) miRNAs were downregulated in three sub-types of diffuse gliomas, which was also corroborated with analysis from The Cancer Genome Atlas (TCGA) datasets. The miRNA expression levels decreased with increasing tumor grade, indicating this downregulation as an early event in gliomagenesis. Higher expression of the C14MC miRNAs significantly improved glioblastioma prognosis (Pearson’s r = 0.62; p < 3.08e-22). ENCODE meta-data analysis, followed by reporter assays validated existence of two novel internal regulators within C14MC. CRISPR activation of the most efficient internal regulator specifically induced members of the downstream miRNA sub-cluster and apoptosis in glioblastoma cells. Luciferase assays validated novel targets for miR-134 and miR-485-5p, two miRNAs from C14MC with the most number of target genes relevant for glioma. Overexpression of miR-134 and miR-485-5p in human glioblastoma cells suppressed invasion and proliferation, respectively. Furthermore, apoptosis was induced by both miRs, individually and in combination. The results emphasize the tumor suppressive role of C14MC in diffuse gliomas, and identifies two specific miRNAs with potential therapeutic value and towards better disease management and therapy.

## Introduction

Glioblastoma (GBM; WHO Grade IV^1^) has a very poor prognosis (median survival of 14 months^2,3^) after decades of research. This highlights the importance of novel alternative approach towards understanding and modulating the molecular pathophysiology of GBM. Regulatory molecules like transcription factors and non-coding RNAs (e.g. microRNAs) are higher order switches that can influence a large number of downstream targets. Deciphering the functioning of these switches will not only enhance our knowledge but also enable us to alter them towards translatable therapy. Understanding the local regulators of such higher order modulators (e.g. promoters of microRNAs) is thus a rational approach to empower such therapeutic potential.

MicroRNAs, the most-studied class of non-coding RNAs, play crucial role in several biological processes, from development to metabolism^4–7^. Several microRNAs have been proposed to possess prognostic importance for patient survival in gliomas^8–10^. Therapeutic interventions involving microRNAs are developing rapidly. A single microRNA has multiple targets, thus they can be used to target entire pathways, although their delivery and stability still remain areas of active research. The role of large microRNA clusters in cancer biology is a relatively new evolving area of research. Chromosome 14 microRNA cluster (C14MC) is located on human 14q32, a locus earlier reported to be unstable in gliomas^11,12^. This second largest miRNA cluster comprises of 73 mature miRNAs, spanning ~45 kb on 14q32.31, within the well-known DLK-DIO3 locus, and is placental mammal lineage specific^13^. The prospect of local regulation within this large cluster is unknown, although the whole domain of DLK1-DIO3 is reported to be under hierarchical interaction via imprinting control regions^14^. C14MC has been implicated in various developmental pathways as well as found to be involved in several conditions, like neurogenesis^5^, neovascularization^15^ and metabolic transition during birth^16^. We have earlier reported overall down-regulation of this cluster in various cancers, including GBM^17^. Individual miRNAs from this cluster have been reported to be deregulated in leukemias^18^, esophageal squamous cell carcinoma^19^, etc. strengthening the speculation that these might be tumor suppressors in several cancer types. However, there are few cases where they are up-regulated, like in lung adenocarcinoma^20^.

Here we demonstrate the tumor suppressor potential of C14MC in GBMs, and evaluate the polycistronic nature of C14MC, hence, opening up new channels for modulating these non-coding elements, towards achieving a desired phenotype. We have assessed differential expression of these miRs in different grades of diffuse astrocytic tumors, which may have latent diagnostic or prognostic utility, when combined with other layers of clinical information. We have also identified key miRNAs from C14MC capable of reducing GBM tumorigenicity and hence may serve as potential future therapeutics.

## Results

### C14MC is downregulated in different grades of astrocytic tumors and higher miRNA expression is associated with better survival in GBM

**Figure 1:**
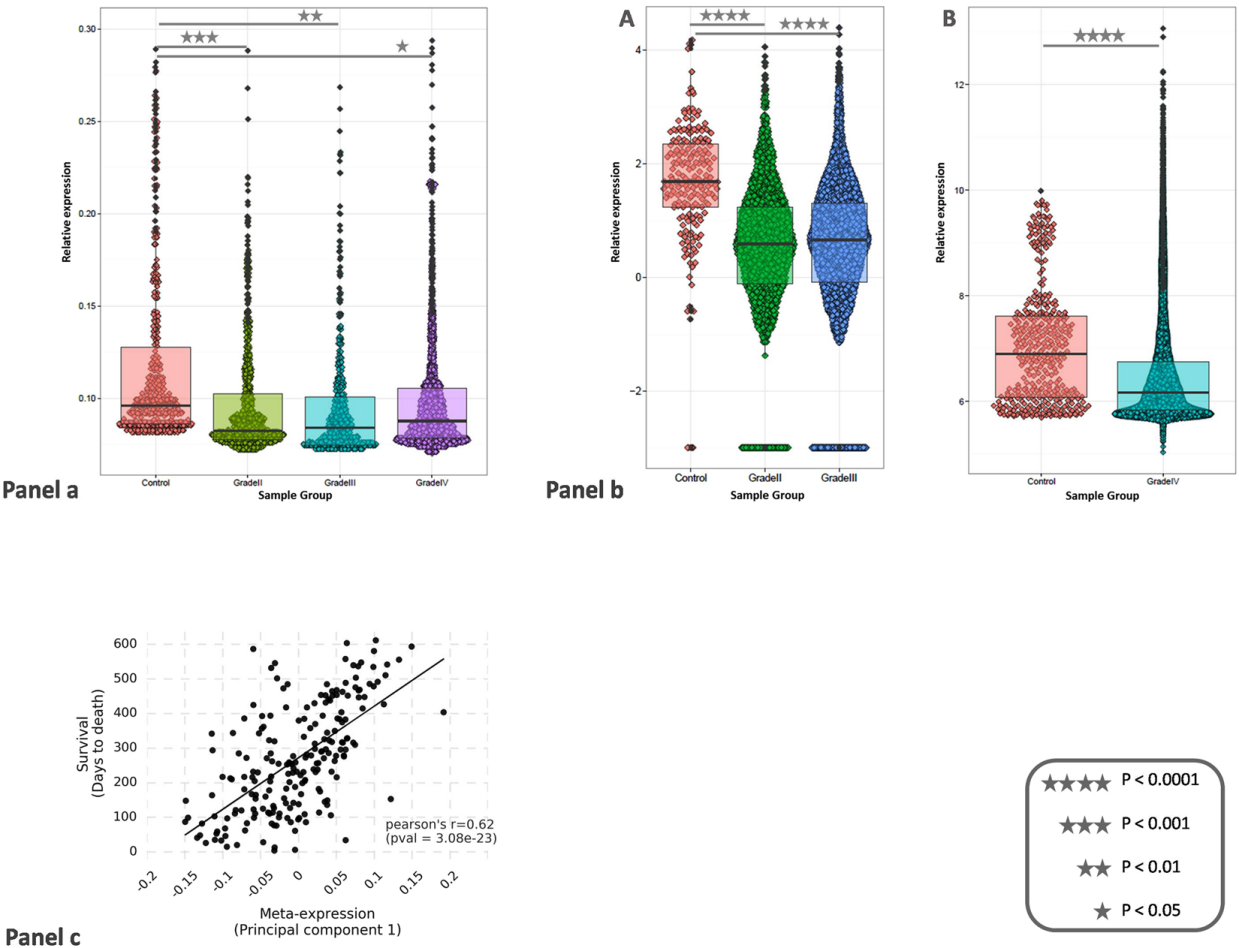
Downregulation of C14MC in gliomas. (**a**) Downregulation of C14MC in gliomas (TLDA data). X-axis shows the group of samples; Grade II (Diffuse astrocytoma; n = 15), Grade III (Anaplastic astrocytoma; n = 9), Grade IV (Glioblastoma; n = 35) and controls (n = 10). Y-axis represents relative expression (delta Ct values). Median expression value of each sample group is represented in the box-plot. (**b**) (A) Downregulation of C14MC in gliomas (TCGA small RNA sequencing data). X-axis shows the group of samples; Grade II (Diffuse astrocytoma; n = 46), Grade III (Anaplastic astrocytoma; n = 97) and controls (n = 5). Y-axis represents relative expression (log transformed). Median expression value of each sample group is represented in the box-plot. (B) Downregulation of C14MC in gliomas (TCGA microarray data). X-axis shows the group of samples; Grade IV (Glioblastoma; n = 505) and controls (n = 10). Y-axis represents relative expression (lowess normalised). Median expression value of each sample group is represented in the box-plot. (**c**) Moderate association of C14MC expression with survival of GBM patients. X-axis represents meta-expression, derived from principal component 1 (extracted from Z-score values of Fold change for each miR across all GBM samples). Y-axis shows survival in days. The association was tested using Pearson’s test, after removing one outlier sample.

Taqman Low Density Array (TLDA; ABI Inc.) was used to evaluate the expression profile of C14MC in diffuse astrocytic tumors. Profiling was done for 47 miRNAs from C14MC in 10 control samples, 35 GBMs (Glioblastoma; Grade IV), nine AAs (Anaplastic astrocytoma; Grade III), and 15 DAs (Diffuse astrocytoma; Grade II). The median number of miRNAs downregulated in GBM, AA and DA were 60%, 87% and 89%, respectively (one tailed Wilcoxon p < 0.05) [Fig. 1a; Supplemental file]. None of the miRs were seen to have significant up-regulation in any of the tumor types as compared to controls. Interestingly, 68% (32/47) miRs were downregulated in all the three tumor types studied, as calculated by 2^−∆∆Ct^ method^21^.

We also used TCGA LGG datasets (Low Grade Glioma) for 148 samples (143 Patients and 5 controls) to validate our findings of lower grade astrocytomas on larger sample size. We identified 37 (95%) and 35 (90%) miRs to be significantly downregulated in both Grade II (n = 46) and III (n = 97) astrocytomas, respectively, as compared to controls even after Bonferroni correction [Fig. 1b], although, we could not find any significant differentially expressing miRs between the two grades. TCGA data analysis revealed ~78% miRNAs downregulated from C14MC in GBM, which is in concordance with our earlier report^17^.

Interestingly, TCGA data (n = 350) revealed significant association of C14MC meta-expression with better survival of GBM patients (Fig. 1c; Pearson’s r 0.62 after removing one outlier sample; p < 3.08e-22). When checked individually, higher expression of 63% (22/35) miRNAs from C14MC was associated with better prognosis of GBM patients (Supplemental file). We found that four miRNAs (miR-134, miR-410, miR-409-3p and miR-494) from C14MC were significantly associated with IDH mutation. IDH-mutated GBM samples showed higher expression of these four miRNAs.

### C14MC is not a single cistron: discovery of internal regulators

**Figure 2:**
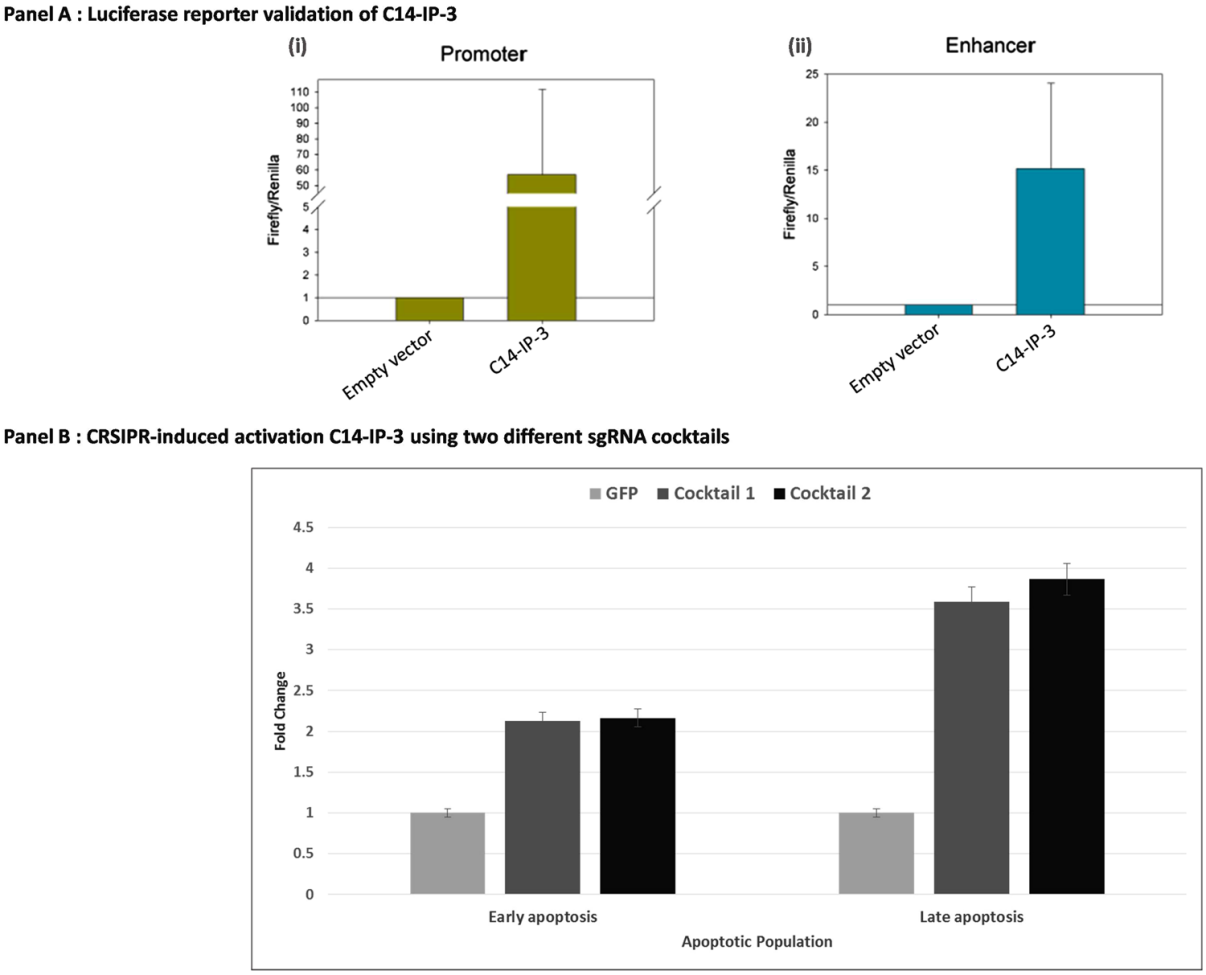
Sub-clusters within C14MC and CRISPR-induced activation of C14-IP-3. Panel A (i): Histogram showing >50 fold higher promoter activity (luciferase reporter) of internal promoter C14-IP-3 as compared to empty vector. Panel A (ii): Histogram showing ~15 fold higher enhancer activity (luciferase reporter) of internal promoter C14-IP-3 as compared to empty vector. X-axis represents the conditions (empty vector and vector with internal promoter), Y-axis shows Firefly/Renilla ratio. Panel B: CRISPR-induced up-regulation of C14-IP-3 results in enhanced apoptosis of glioma cells. X-axis shows early and late apoptotic cell population. Y-axis shows the fold change of apoptotic population for all the transfection conditions, in comparison to control. The fold change was checked in two different cocktails of sgRNAs designed against C14-IP-3 (3 sgRNAs in each cocktail), in comparison to sg_GFP transfection. The variations within replicates is indicated by 5% error bars.

We next investigated the polycistronic nature of C14MC for which integration of different layers of data, namely RNA-Seq and the presence of various histone marks, representative of transcriptionally active and repressed regions were used from ENCODE HUVEC and NHA datasets, respectively. It enabled better documentation of potential regulators in the ~60 kb region of C14MC (including ~20 kb upstream of the cluster), in view of the normal cell population. This would be representative of the physiological condition of the chromatin and the transcriptome contributed by this locus. Superimposition of epigenetic and transcriptomic data from ENCODE revealed four regions, having regulatory potential, in and around C14MC (Supplemental file). One of them, named Chromosome 14 Internal Promoter 3 (C14-IP-3) showed enhanced activity compared to empty vector [Fig. 2A], both as promoter (>50 fold increased activity) and as enhancer (>15 fold increased activity).

Since this promoter showed consistently robust activity, in contrast to C14-IP-1, C14-IP-2 and C14-IP-4, we selected it for further downstream studies.

### Positive induction of C14-IP-3 increases apoptosis in glioma

We next investigated the role of C14-IP-3 upon the apoptotic potential of glioma cells, using a CRISPR activation (CRISPRa) approach. For this, we used a catalytically inactive form of Cas9 (dCas9) fused with an activating domain (VP64) to selectively upregulate miRNAs from the C14-IP-3 sub-cluster. Using this approach, we could obtain between 1.2–2.5 fold upregulation of five out of eight sub-cluster miRNAs tested (Supplemental file). Interestingly, upon overexpression of these miRNAs, GBM cells showed ~3.6 fold and 2 fold increase respectively, in total and early apoptotic cell population [Fig. 2B; Supplemental file]. Taken together, this suggests the possibility of modulating the internal regulator in order to gain a desired expression pattern of the subcluster downstream – thus enabling a reversal of phenotype.

### Key miRNAs from C14-IP-3 sub-cluster target EGFR, AKT, TP53 and RAF1 in GBM

**Figure 3:**
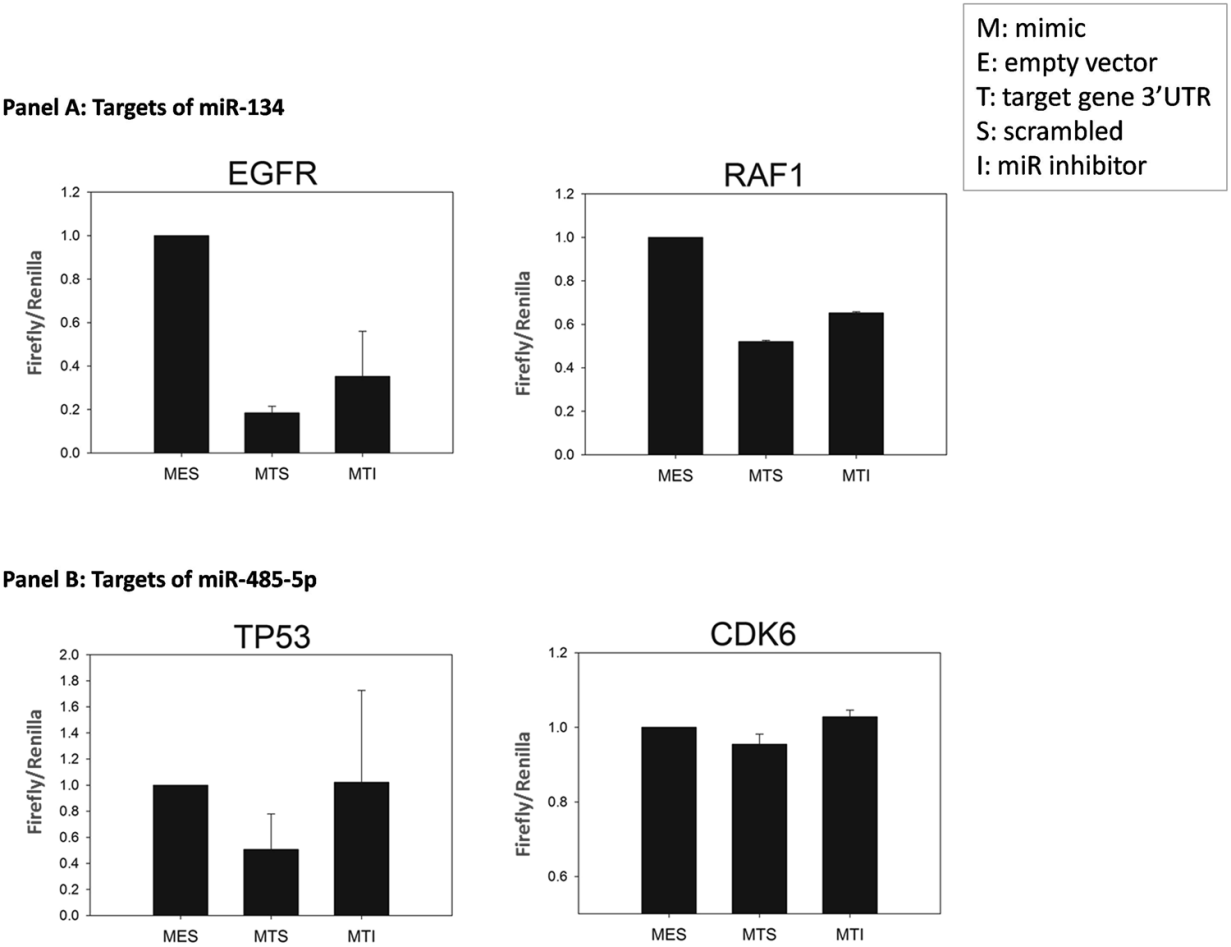
Target validation of miR-134 and miR-485-5p. Panel A: Histogram showing luciferase reporter based miR-mRNA interaction of miR-134 with EGFR and RAF1. Panel B: Histogram showing luciferase reporter based miR-mRNA interaction of miR-485-5p with TP53, and negligible evidence of CDK6 interaction. X-axis represents the conditions (MES: miR mimic + empty vector + scrambled; MTS: miR mimic + target gene UTR + scrambled; MTI: miR mimic + target gene UTR + miR inhibitor), Y-axis shows Firefly/Renilla ratio. Error bars indicate standard error.

We chose miR-134 and miR-485 from C14-IP-3 as they were predicted to target the highest number of targets in the “glioma” pathway^17^. In this study these two miRNAs are mentioned as ‘Key’ miRNAs highlighting their possible crucial roles in modulating biological outcomes. Consequently, we experimentally validated three out of four predicted downstream targets of these miRNAs, namely EGFR, TP53 and RAF1 – all known for their importance in glioma biology (Fig. 3A,B).

EGFR and RAF1 are novel targets of miR-134, which were successfully validated by luciferase assay. Additionally, we identified TP53 as a novel target of miR-485-5p, and validated it. However, we could not find positive interaction of CDK6 with miR-485-5p.

We randomly selected four additional genes (AKT2, AKT3 GRB2 and MMP2) from the “glioma” pathway, other than the validated targets of the key miRNAs.to check for downstream effect of miRNA overexpression. Five out of seven genes tested showed downregulation at the RNA level upon miR-134 overexpression (EGFR, RAF1, AKT2, TP53 and MMP2), whereas, two (MMP2 and RAF1) shared similar fate after miR-485 overexpression [Supplemental file]. EGFR, AKT, FAK and TP53 were also checked at protein level upon miRNA overexpression [Supplemental file]. The protein levels of EGFR and AKT showed about 10% decrease upon overexpression of both the miRNAs, whereas, TP53 showed ~8% decrease upon miR-485-5p overexpression. Interestingly, the most dramatic decrease (~75%) was seen in pAKT levels upon miR-485-5p overexpression.

### MiR-134 overexpression decreases GBM cell invasion

**Figure 4:**
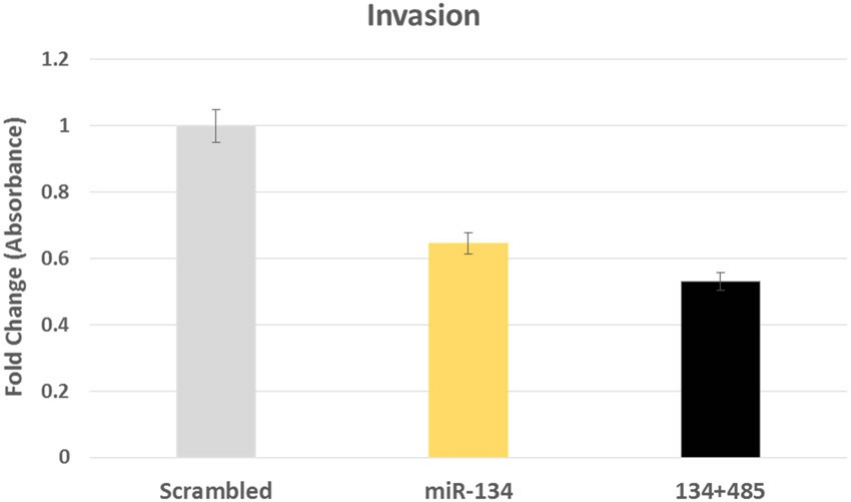
Invasion suppression by miR-134 and miR-485-5p. Histogram showing ~30% decrease of invasiveness upon miR-134 transfection. This effect is dramatically increased upon transfection of both miR-134 and miR-485-5p, which resulted in ~50% decrease in invasion of glioma cells. X-axis represents the transfection conditions, Y-axis shows proportion of cell population. Variation in the triplicates is shown as 5% error bars.

Matrigel assay consistently revealed an average of ~30% decrease in cell invasion, after 48 hours of overexpression of miR-134 in U87-MG cells, compared to scrambled oligo transfection. We also found minimal decreased invasion in miR-485-5p transfected cells, but the data remained inconclusive. Cumulative effect of the miRNAs was also checked, using an equimolar mixture of both the miRs, which interestingly resulted in ~50% decrease in invasiveness of U87MG cells [Fig. 4; Supplemental file].

### MiR-485-5p overexpression blocks GBM cell proliferation

The effect of overexpression of both the miRNAs on cell proliferation of U87 cells, 48 hours post transfection, was striking (~50% decrease in case of miR-134 and ~75% decrease in case of miR-485) compared to normal cells. A comparison with scrambled transfection showed a statistically significant decrease in proliferation only for miR-485-5p.

BrdU assay revealed ~40% decrease in cell proliferation, upon overexpression of miR-485-5p in glioma cell line, compared to the scrambled, evident from the decreased S-phase population of cells upon miR-485-5p transfection. The results for overexpression of miR-134 and combination (miR-134 + miR485-5p) were negligible.

**Figure 5:**
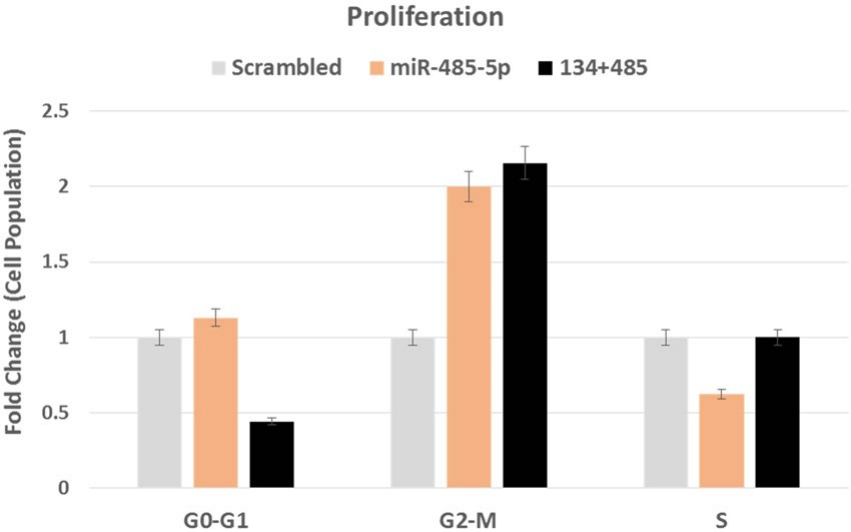
G2-M arrest by miR-134 and miR-485-5p. Histogram showing 2 fold increase in G2-M population, with a ~40% decrease in S-phase cells upon miR-485-5p transfection. This effect is dramatically increased upon transfection of both miR-134 and miR-485-5p, which resulted in ~2.2 fold increase in G2-M phase cells. X-axis represents the transfection conditions, Y-axis shows proportion of cell population. Variation in the triplicates is shown as 5% error bars.

We also observed a G2-M phase block after 24 hours of transfection of miR-485-5p, evident from increased G2-M population of cells (2 fold increase). When the cells were treated with a combination of both the miRNAs, a similar degree (2.2 fold) of G2-M phase arrest was noted [Fig. 5; Supplemental file].

### Overexpression of key miRNAs make GBM cells pro-apoptotic

We found a substantial amount (from 45% in scrambled transfected cells to 31% in miR-134 transfected cells) of the cell population to be in the viable section for all the transfected conditions, and moderate changes in the total apoptotic cell population. However, there was ~1.4 fold higher cells in the early apoptotic section upon miR-134 overexpression, and a similar trend of 1.3 fold higher upon miR-485-5p transfection compared to scrambled transfection. The changes seen in the late apoptotic population of cells were very moderate.

**Figure 6:**
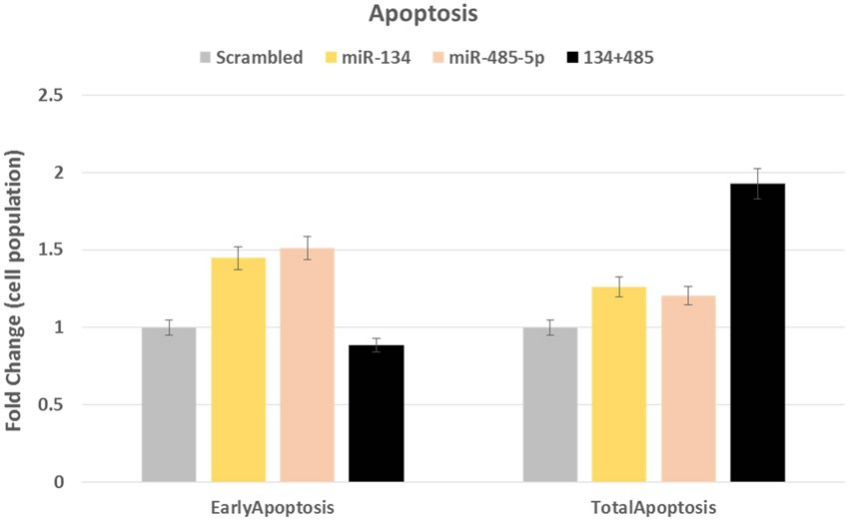
Apoptosis induction by miR-134 and miR-485-5p. Histogram showing ~1.5 fold increase in early apoptotic cell population upon either miR-134 or miR-485-5p transfection, and moderate increase in total apoptotic cell population. This effect is dramatically increased upon transfection of both miR-134 and miR-485-5p, which resulted in ~2 fold increase in total apoptotic cell population, with not much change in early apoptotic cells. X-axis represents the transfection conditions, Y-axis shows proportion of cell population. Variation in the triplicates is shown as 5% error bars.

Interestingly, when the cells were treated with a mixture of both the miRNAs, a marked increase (~2 fold) in total apoptotic cell population was observed, whereas the early apoptotic cell population was negligible in miR-overexpressed cells, compared to scrambled transfection [Fig. 6; Supplemental file]. This suggests the tumor suppressive potential of both of the miRNAs in GBM.

## Discussion

In glioblastoma, the higher-order, epigenetic regulatory components such as chromatin regulators, transcription factor alterations and non-coding genome aberrations play equally important roles in the tumorigenesis and progression of cancer^22,23^, as that of spontaneous somatic alterations and chronological selection for aggressive clones^24,25^. MicroRNAs, the most widely studied class of non-coding RNAs, have been reported to regulate various cellular and developmental processes^26–28^. Besides, deregulation of miRNAs is a common observation in numerous pathological conditions, including cancer, metabolic disorders, CNS disorders and cardiovascular diseases^29–31^ - implicating their wide potential in therapeutic intervention.

Studies focussed on this miRNA cluster (C14MC; miR-379/miR-656 cluster) show its deregulation in various cancers, including gliomas^11,12^. In this study we show that C14MC downregulation is also consistent in other subtypes of diffuse astrocytic tumors. The median number of miRs downregulated in GBM was lower (60%) compared to the lower grades (>85%), which are the early stages of glioma. A greater extent of downregulation in the early stages indicated the downregulation to be an early event in the disease biology (Fig. 1), seen in both TCGA and in-house datasets. The systematic downregulation also indicates that one or more of the regulatory units for C14MC and cross-talks between them is altered in gliomas. This observation is also in agreement with an earlier report on another low grade glioma, medulloblastoma^32^. Patients with IDH mutations have been reported to have a better prognosis compared to those without such a mutation^1^. Interestingly, four C14MC miRNAs show higher expression in IDH-mutated GBM samples, suggestive of their prognostic value. Overall, higher expression of >60% of C14MC members was associated with better prognosis (survival) of GBM patients [Supplemental file]. We also used meta-expression of C14MC as a whole and found a significant association of higher expression with better survival [Panel C of Fig. 1]. Thus it was evident that a potentially better management of the disease is possible, if we can increase the level of expression for C14MC as a whole or for majority of its members.

Hierarchical interaction of imprinting control regions have been reported for the whole domain of Dlk1-Dio3^14^, containing C14MC, but the probability of local regulation within C14MC was not attempted. We wanted to decipher the cis-regulators for C14MC further, to alter the state of multiple miRNAs simultaneously. As shown in supplemental file, we identified potential internal regulators by superimposing multiple layers of genetic and epigenetic information, as earlier reported by Ozsolak *et al*.^33^. The analysis indicated at least three different cis-regulatory regions within the genomic region of C14MC. We experimentally validated the presence of sub-clusters along with two internal cis-regulators within C14MC (Fig. 2A). Functional studies revealed varied potential of these regulators and we focused on the most potent cis-regulator (Fig. 2A) with 20 miRNAs downstream. The discovery of these internal cis-regulators is one of the most important findings of this study as this will allow selective modulation of sub-set of miRNAs, in turn modulate a pathway.

We utilised a CRISPR-cas9 based promoter induction system to test our hypothesis of altering the cancerous phenotype of human GBM cells by increasing the expression of specific miRNAs. CRISPR mediated activation approaches have been reported to be efficient for gene activation in multiple reports^34–36^. Activation of C14-IP-3 using CRISPR-cas9 system resulted in increased expression of the specific miRNAs under its control and increased apoptosis of GBM cells (Fig. 2B). This is in direct agreement with our data on improved patient survival with increased miRNA expression for C14MC members.

We earlier reported a possible coordinated regulation of the ‘glioma’ pathway by members of C14MC (Fig. 1 in^17^). Amongst various miRNAs, miR-134 (eight targets) and miR-485-5p (six targets) were predicted to target the maximum number of genes in the ‘glioma’ pathway^17^. These miRNAs were our natural choice to test as key miRNAs for their relevant multiple targets as well as for the fact that both of them were under the control of C14-IP-3, the most efficient internal promoter. MiR-134 is a brain-specific microRNA and already have proven roles in hippocampal neurons^37^ and also in differentiation of embryonic stem cells^38^. This miRNA has also been reported to inhibit epithelial to mesenchymal transition in lung cancers^39^, as well as identified as a metastasis inhibitor in hepatocellular carcinoma^40^. MiR-485-5p has been found to be differentially expressed in various cancers, including leukemia^41^ and lung^42^ cancer. Thus these two miRNAs together are capable of targeting vital cellular processes like cytokine-cytokine receptor interaction, ErbB signalling, MAPK signalling, calcium signalling, mTOR signalling and cell cycle regulation^17^, which are known to be dysregulated in GBM.

We validated EGFR and RAF1 as direct targets of miR-134 via luciferase assay. Recently, Forloni *et al*., showed that oncogenic *EGFR* blocks tumor suppressor genes, inhibiting *TET1* in lung cancer and GBM^43^. RAFs are well studied oncogenes. They have been reported to be overexpressed in malignant gliomas^44^ and have thus been targeted for treatment^45^. Activated RAF/RAS pathway leads to activated AKTs, which have been reported to be essential for glioblastoma maintenance *in vivo*^46^. A recent report claimed that the combined blockage of Akt/mTOR and MDM2 augments cell apoptosis and differentiation in GBM cancer stem cells^47^. Our finding of specific miRNAs targeting these molecules provides more ways of altering the gene expressions for a positive outcome.

TP53 was also validated to have direct interaction with miR-485-5p in this study. *TP53* has been classically designated as the guardian of the cell, protecting it from uncontrolled growth, inducing DNA repair and apoptosis, in response to oncogenic stimuli^48,49^. But, evidences in recent literature suggest mutated TP53 promoting tumorigenesis^50,51^. Based on our results, miR-485-5p can have tumor-suppressor roles, by targeting and downregulating the mutant TP53, as the targeting region in the 3′-UTR is identical between the wild-type and the mutant.

Overexpression of these two miRNAs in glioma cells revealed blockage of cell invasion and proliferation, along with G2-M cell phase arrest, as well as increase in apoptotic cell population. When used in combination these effects were amplified. Thus, we propose that these two miRs should be considered for future therapeutics of GBM as they can tackle three characteristic features of glioblastoma namely, invasiveness, aggressive cell proliferation and evasion of cell cycle arrest.

MicroRNA therapeutics as a field is emerging fast, enabling researchers and pharmaceutical companies across the globe to identify potential miRNAs and move onto preclinical and clinical stages of miRNA-based therapeutics. There have been reports of miRs inhibiting cell proliferation^52,53^ and inhibiting migration^53,54^ in various cancers. Our study thus becomes one of the first such studies in glioma to suggest novel cis-regulators for a very relevant set of miRNAs and also we recommend two specific miRNAs to be studied for their therapeutic potential. Furthermore, the key miRNAs, miR-134 and miR-485-5p show the tumor suppressive potential for GBM and reveal novel therapeutic potential for glioma. How these sub-clusters and their regulators are in cross-talk with the overall regulation of this imprinted region will be a very interesting question to be addressed in future research.

## Materials and Methods

### Cell Lines, Patient and Control tissue specimens

A total of 35 GBM (glioblastoma multiforme, Grade IV), 9 AA (anaplastic astrocytoma, Grade III), 15 DA (diffused astrocytoma, Grade II), and 10 control samples were taken for the study. All tumor samples were collected after informed consent was obtained in writing. All the methods followed in this study were in accordance with the Helsinki Declaration and the ethical board of All India Institute of Medical Sciences, Delhi, India approved all the methods included in the project. The control samples included in this study have been obtained from the NIMHANS brain bank, Bangalore, India, and include normal human corpus callosum tissues from road accident victims, without head injury. Fresh frozen samples were used for nucleic acid isolation. Flanking sections of the sample, measuring 5 micron were taken and stained by H&E for histological analysis. The histopathology of each collected specimen was reviewed to confirm adequacy of the sample (i.e, minimum contamination of non-neoplastic elements) and to assess the extent of tumoral necrosis and cellularity. *In vitro* experiments were conducted on U87-MG cell line and LN229.

### RNA Isolation and Expression profiling of C14MC (TLDA)

The total RNA extraction for all the samples under discussion was done by miRVana miRNA isolation kit (Ambion), using the manufacturer’s protocol. The total RNA samples were processed further for cDNA conversion. Taqman assay for microRNAs was done using Taqman microRNA Reverse Transcription Kit (Life Technologies), followed by customized Taqman Low Density Arrays (TLDA) to quantitate the levels of 47 microRNAs from the chromosome 14 microRNA cluster, as per manufacturer’s protocol (we could find readily available assays for these miRs only). Quantitative PCR was conducted in a 10 µL volume on 7900HT Fast Real-Time PCR System. Ct values greater than 35 were considered as 35 for further analysis. The relative quantification was calculated by 2^−∆∆Ct^ method^21^. Differentially expressed miRs were selected according to the fold change more than 2 (for up-regulation) and less than 0.6 (for down-regulation). Further details of the methodology are given in Supplemental file.

### Survival analysis and association with *IDH* mutation from TCGA

Level 3 miRNA expression data and corresponding clinical information for GBM samples were taken from TCGA. Hazard curves for each miRNA were calculated using Kaplan-Meier survival function estimation method as implemented in Python package Lifelines. For calculation of survival function the samples were divided into two groups based on expression of the given miRNA. The partitioning were done such that samples with expression values above 80th percentile were placed in one group and those below 20th percentile were placed in the other group. The significance of difference between survival functions of the two groups was assessed using log rank test. The samples with survival more than 730 days were censored. Plotting of hazard curves and their 95% confidence interval was done using custom Python scripts. Principal component analysis was performed using Python’s Scipy package.

IDH being the major classifier in the recent WHO classification, its association with C14MC expression was tested, to check for any significant difference of C14MC expression between IDH mutant and IDH wildtype GBM samples (n = 211; 13 mutant and 198 wildtype). Somatic variation datasets (level 3) were obtained from TCGA for *IDH*. This data was used for association testing with C14MC miRNA expression for the same samples (using level 3 miRNA microarray expression data). Statistical significance for association between both datasets was tested using student’s t-test (assuming unequal variance), with a significance threshold of 0.05.

### Prediction of local regulators

Epigenetic marks known for transcriptionally active regions (H3K4me1, H3K4me2, H3K4me3, H3K27ac, H3K9ac), and those known to be found in repressed regions (H3K27me3, H4K20me1) were checked in the NHA (normal human astrocytes). This data was superimposed with that of the contigs built from polyA+ RNA Seq data from HUVEC (method details given in Supplemental file), performed on nuclear fraction. The regions where the epigenetic marks and the breaks in contig overlapped were predicted to harbour potential regulatory role. All the data used were from ENCODE (GSE30567), and visualized in the UCSC Genome Browser (hg19).

### Cloning of C14-IP3 and luciferase assay

Genomic DNA served as template for PCR amplification for the probable regulator, prior to cloning into pgl3-Basic and pgl3-Promoter plasmids (details given in Supplemental file). Glioma cell lines (U87-MG) were seeded in a 12-well plate using Lipofectamine 3000 (Invitrogen), using the manufacture’s protocol, when the cells reached a confluency of ~80%. 10 ng Renilla plasmid was used as the transfection control. The ratio of Lipo to DNA used was 1:3 (1 ug DNA was used). The conditions used were lipo-only, empty vector, plasmid with the regulatory region inserted. Twenty-four hours post transfection, cells were washed twice with 1x PBS, and lysed with 80 µL of 1x passive lysis buffer (Promega), followed by the Dual luciferase reporter activity assay, according to the manufacturer’s protocol (Promega), using Infinite M200 (Tecan). Data was expressed as means ± standard errors of replicates.

### CRISPR-Cas9 modulation of local regulator

The genomic sequence of C14-IP-3 was obtained from UCSC Genome Browser, and was subjected through sgRNA predicting software of CRISPRSCAN, with information on human genome off-targets. A total of 10 sgRNAs could be designed against C14-IP-3. We have used a catalytically inactive form of Cas9 (dCas9) and fused it with an activating domain (VP64) to bring about the CRISPR activation (CRISPRa). As C14 miRNAs are downregulated in glioblastoma, CRISPRa approach was used to induce upregulation of C14-IP-3. This was attained by using two different cocktails, comprising of three sgRNAs each. Transfection of sgGFP was done, to serve as control. To assess the effect of this modulation, differential expression of randomly selected several miRNAs from the genome and the sub-cluster was studied. RNA isolation and Real time PCR protocol has been given in Supplemental file. Two different cocktails were chosen to assess the impact on cell phenotype (apoptosis) upon CRISPR-induced upregulation of C14-IP-3. Generation and purification of sgRNAs, and transfection details of CRIPSR-induced modulations are given in Supplemental file. The details of apoptosis assay can be found in subsequent methods section. The CRISPRa was done using Lipofectamine 3000 in glioma cells (LN229).

### miR-mRNA interaction

As we earlier reported, miR-485-5p and miR-134 target important genes in the “glioma” pathway^17^. These two miRs from C14MC target the maximum number of genes in the pathway. Four predicted targets were taken for validation using luciferase assay in glioma cells (U87-MG), using 3′UTR clones from Genecopoeia (USA) and miR mimics from Dhermacon (GE-Dhermacon Inc., USA). The detailed methodology of the luciferase assay and subsequent relative expression of target genes upon miR overexpression has been given in the supplemental file.

### Invasion assay

Glioma cells (~50,000) were seeded onto the matrigel chamber insert with serum free media, 24 hours post transfection of the miRs. The BioCoat Matrigel plates used in the study were procured from BD Biosciences, and were of 24-well format. After 48 hours of miR mimic transfection, the media was removed from both the chambers. The inside of the upper chamber was gently wiped with 70% ethanol to remove any cell attached to the inner portion of the matrigel, followed by staining the chamber in 0.2% crystal violet, incubating it at room temperature for ~20 mins. The chambers were then washed with milliQ water and air dried for ~20 mins. After taking images of the stained chamber inserts, they were put in 1x cell lysis buffer for ~20 mins at room temperature, and protein absorbance was taken at 562 nm using Infinite M200 (Tecan). This assay was done in five replicates.

### Proliferation assay

Proliferation assay was done using BrdU incorporation in glioma cells 48 hours post miRNA transfection. BrdU diluted to 1x (final conc. of 10 µM) in media was added to the cells 6 hours prior to completion of 24 hours of transfection. The cells were then harvested (via Accutase, Thermo Fischer Scientific) and the assay was performed using APC BrdU Flow kit (BD Pharmigen) as per the manufacturer’s protocol. The cells were analysed using BD FACSAria, acquiring 10,000 events per condition. The assay was done in triplicates. Four assay controls (untreated cells with no staining, all stained cells, BrdU untreated cells but with staining, BrdU treated cells but unstained with APC) were used to ensure the proper gating during analysis.

### Apoptosis Assay

Annexin V (FITC labelled) and PI were (BD Pharmigen) used for this assay. Glioma cells transfected with miRNA mimics and inhibitors were harvested (via Accutase, Thermo Fischer Scientific) after 48 hours and subjected to the apoptosis assay as per the manufacturer’s protocol. The cells were analysed using BD FACSAria, acquiring 10,000 events per condition. The assay was done in triplicates. Apoptosis was induced in assay controls by treating cells with 0.1% H_2_O_2._ Four assay controls (untreated cells with no staining, treated cells stained with PI only, Annexin V only, and both) were used to ensure the proper gating during analysis.

### Statistical Analysis

Wilcoxon test was used for significance testing of differential expression in different grades, using R statistical package. Kaplan-Meier analysis was performed using Scipy package in Python (details in Supplemental file). A significance level of p < 0.05 was considered significant for all the tests.

## Electronic supplementary material

Supplementary information
