# Supplementary material for: Novel internal regulators and candidate miRNAs within miR-379/miR-656 miRNA cluster can alter cellular phenotype of human glioblastoma
Source: Sci Rep. 2018 May 16;8:7673. doi: 10.1038/s41598-018-26000-8 (PMC5955984; doi:10.1038/s41598-018-26000-8)
Supplement: Supplementary file 1 — Supplementary information [file 41598_2018_26000_MOESM1_ESM.pdf]

**Novel internal regulators and candidate miRNAs within miR-379/miR-656 miRNA cluster can alter cellular phenotype of human glioblastoma**

Subhashree Nayak[1], Meghali Aich[2], Anupam Kumar[3], Suman Sengupta[4], Prajakta Bajad[2], Parashar Dhapola[5,6], Deepanjan Paul[2,6], Kiran Narta[2,6], Suvendu Purkrait[7], Bharati Mehani[2,6], Ashish Suri[3], Debojyoti Chakraborty[2,6], Arijit Mukhopadhyay[2,6,8]*, Chitra Sarkar[1]*

[1] Department of Pathology, All India Institute of Medical Sciences, New Delhi 110029, India
[2] Genomics and Molecular Medicine Unit, CSIR-Institute of Genomics and Integrative Biology, Mathura Road, New Delhi 110020, India
[3] Department of Neurosurgery, All India Institute of Medical Sciences, New Delhi 110029, India
[4] Proteomics and Structural Biology Unit, CSIR-Institute of Genomics and Integrative Biology, Mathura Road, New Delhi 110020, India
[5] G.N.Ramachandran Knowledge Centre for Genome Informatics, CSIR-Institute of Genomics and Integrative Biology, New Delhi 11020, India.
[6] Academy of Scientific and Innovative Research (AcSIR), Delhi, India.
[7] Department of Pathology, All India Institute of Medical Sciences, Bhubaneswar 751019, Odisha, India
[8] School of Environment and Life Sciences, University of Salford, Manchester, United Kingdom

***Corresponding Authors**

Dr. Arijit Mukhopadhyay
School of Environment and Life Sciences
University of Salford, Manchester, United Kingdom
Phone: +44(0)1612958129
E-mail: a.mukhopadhyay@salford.ac.uk or arijit.mukhopadhyay@gmail.com

Prof. Chitra Sarkar
Department of Pathology, All India Institute of Medical Sciences, New Delhi, India
Phone: +91-11-26593371 Fax: +91-11-26588663, +91-11-26588641
Email: sarkar.chitra@gmail.com

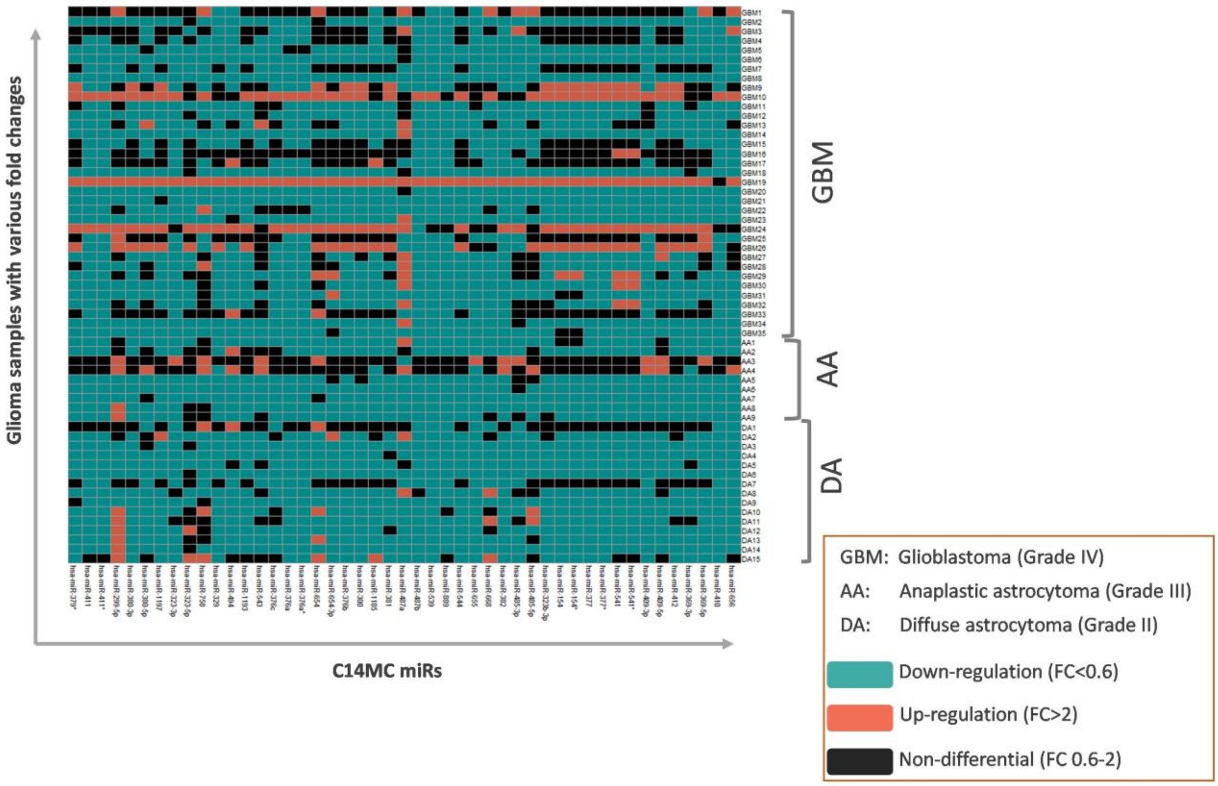


Heatmap showing differential expression (fold change) of C14 miRNAs in different grades of astrocytic tumours. X-axis is the C14MC miRNAs, positioned as they occur in the genome in 5' to 3' direction; samples are arranged vertically (35 GBMs, 9 AAs and 15 DAs). The fold change is calculated against 10 control samples and normalized with U6 SiRNA as the internal control.

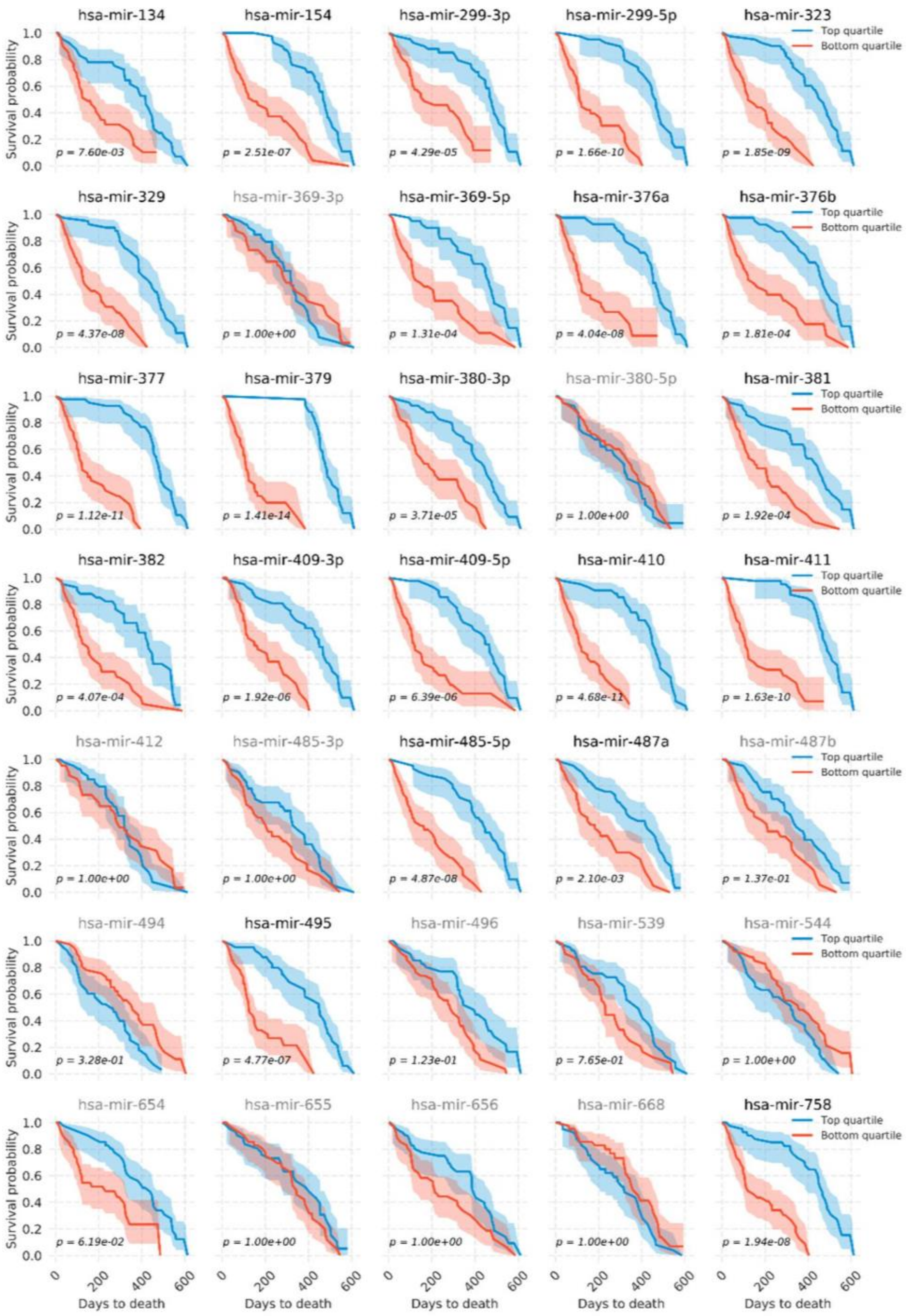


Kaplan-Meier plots for all the miRNAs in C14MC. Higher expression levels for majority (63%, 22/35 after Bonferroni correction) of these miRNAs is associated with better survival in GBM samples (Data source: TCGA datasets). MiRNAs in bold are statistically significant

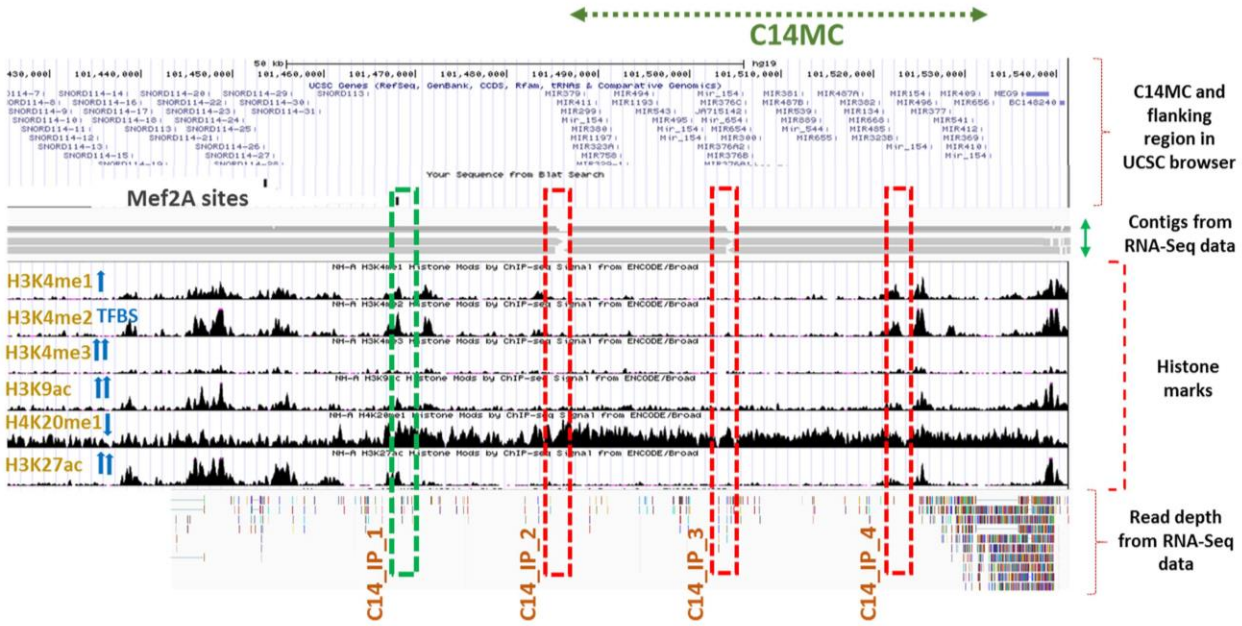


Integration of epigenomic and transcriptomic data from ENCODE for NHA cells reveal probable local regulators. The four regulatory regions have been marked in dotted red lines, which have been named as follows: C14_IP_1 to C14_IP_4 (Chromosome 14 Internal Promoter 1 to 4).

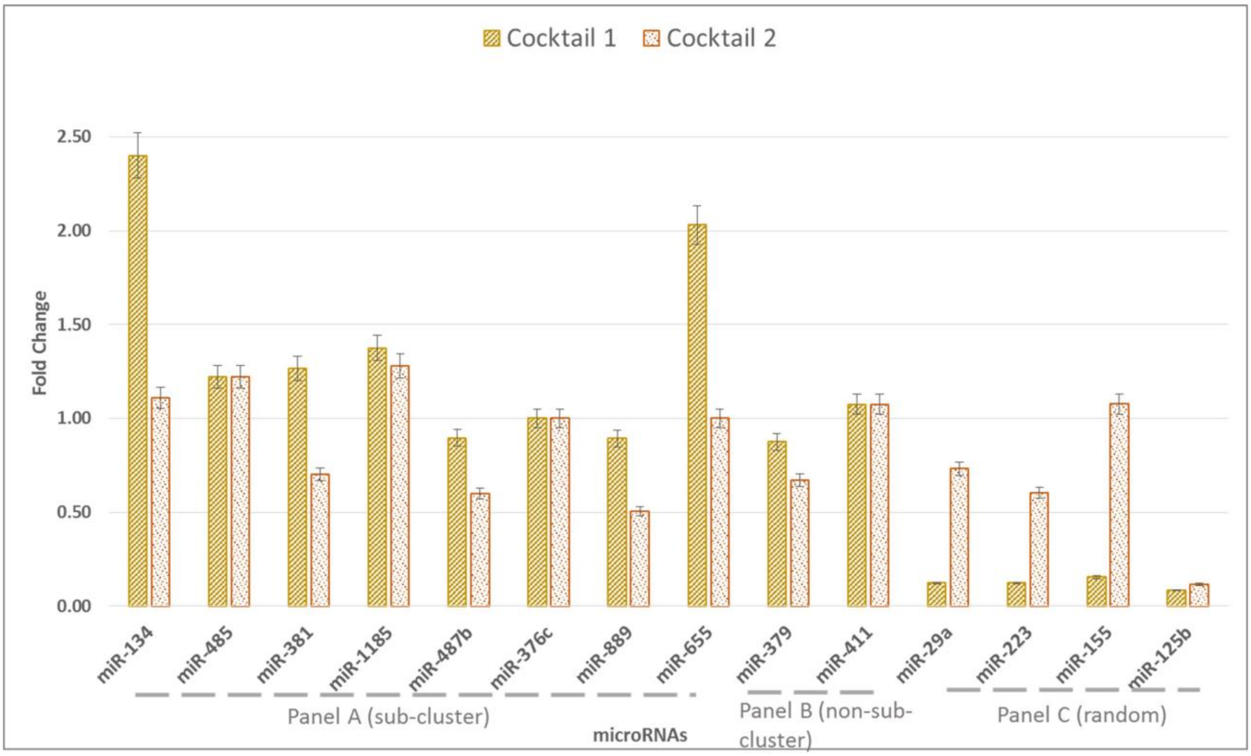


Real Time PCR data upon CRISPRa of C14-IP-3 (PDF 93 kb). Fold Change plotted, as compared to sgGFP transfection (using U6 snRNA as internal control), upon CRISPR-induced up-regulation of C14-IP-3 in glioma cells. X-axis shows different miRNAs (Panel A: C14MC miRs downstream of C14-IP-3; Panel B: C14MC miRs upstream of C14-IP-3; Panel C: miRNAs not from C14MC). Y-axis shows fold change of each miRNA as compared to sgGFP transfection. The fold change was checked in two different cocktails of sgRNAs designed against C14-IP-3 (3 sgRNAs in each cocktail). The variations within replicates is indicated by 5% error bars.

# GFP

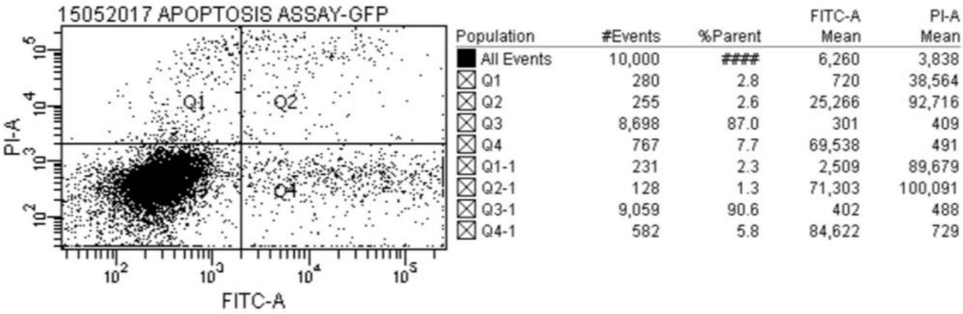


| Population | #Events | %Parent | FITC-A Mean | PI-A Mean |
|---|---|---|---|---|
| All Events | 10,000 | #### | 6,260 | 3,838 |
| Q1 | 280 | 2.8 | 720 | 38,564 |
| Q2 | 255 | 2.6 | 25,266 | 92,716 |
| Q3 | 8,698 | 87.0 | 301 | 409 |
| Q4 | 767 | 7.7 | 69,538 | 491 |
| Q1-1 | 231 | 2.3 | 2,509 | 89,679 |
| Q2-1 | 128 | 1.3 | 71,303 | 100,091 |
| Q3-1 | 9,059 | 90.6 | 402 | 488 |
| Q4-1 | 582 | 5.8 | 84,622 | 729 |

# Cocktail 1

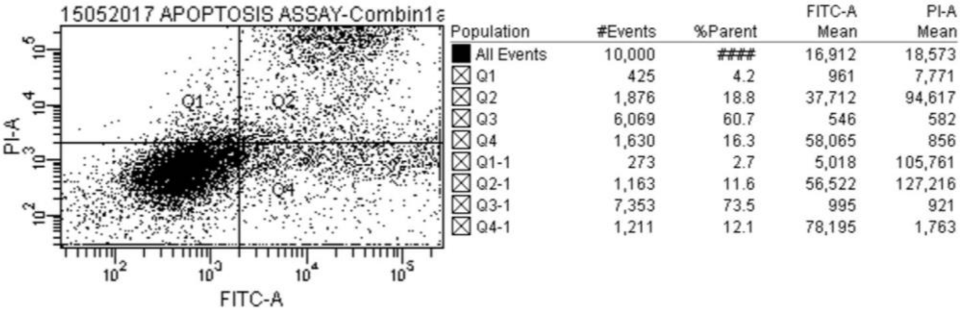


| Population | #Events | %Parent | FITC-A Mean | PI-A Mean |
|---|---|---|---|---|
| All Events | 10,000 | #### | 16,912 | 18,573 |
| Q1 | 425 | 4.2 | 961 | 7,771 |
| Q2 | 1,876 | 18.8 | 37,712 | 94,617 |
| Q3 | 6,069 | 60.7 | 546 | 582 |
| Q4 | 1,630 | 16.3 | 58,065 | 856 |
| Q1-1 | 273 | 2.7 | 5,018 | 105,761 |
| Q2-1 | 1,163 | 11.6 | 56,522 | 127,216 |
| Q3-1 | 7,353 | 73.5 | 995 | 921 |
| Q4-1 | 1,211 | 12.1 | 78,195 | 1,763 |

# Cocktail 2

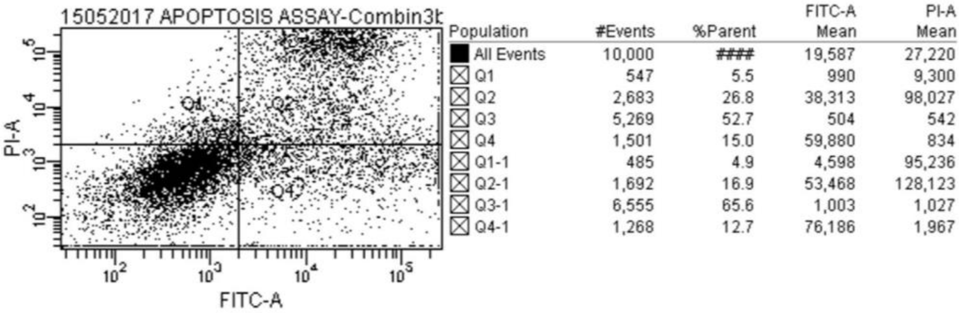


| Population | #Events | %Parent | FITC-A Mean | PI-A Mean |
|---|---|---|---|---|
| All Events | 10,000 | #### | 19,587 | 27,220 |
| Q1 | 547 | 5.5 | 990 | 9,300 |
| Q2 | 2,683 | 26.8 | 38,313 | 98,027 |
| Q3 | 5,269 | 52.7 | 504 | 542 |
| Q4 | 1,501 | 15.0 | 59,880 | 834 |
| Q1-1 | 485 | 4.9 | 4,598 | 95,236 |
| Q2-1 | 1,692 | 16.9 | 53,468 | 128,123 |
| Q3-1 | 6,555 | 65.6 | 1,003 | 1,027 |
| Q4-1 | 1,268 | 12.7 | 76,186 | 1,967 |

Representative image of AnnexinV-PI assay showing increased apoptosis upon administration of different combinations of sgRNAs designed against the genomic locus of C14-IP-3 in glioma cells, as compared to sg_GFP.

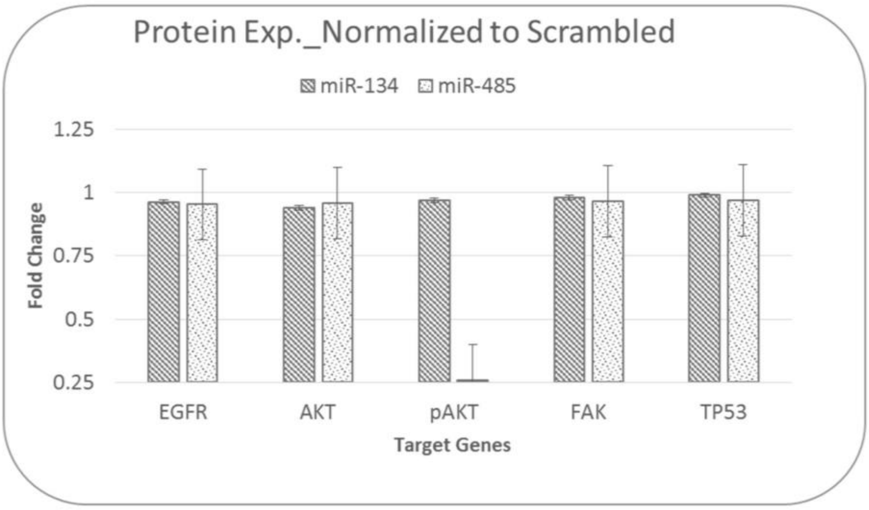


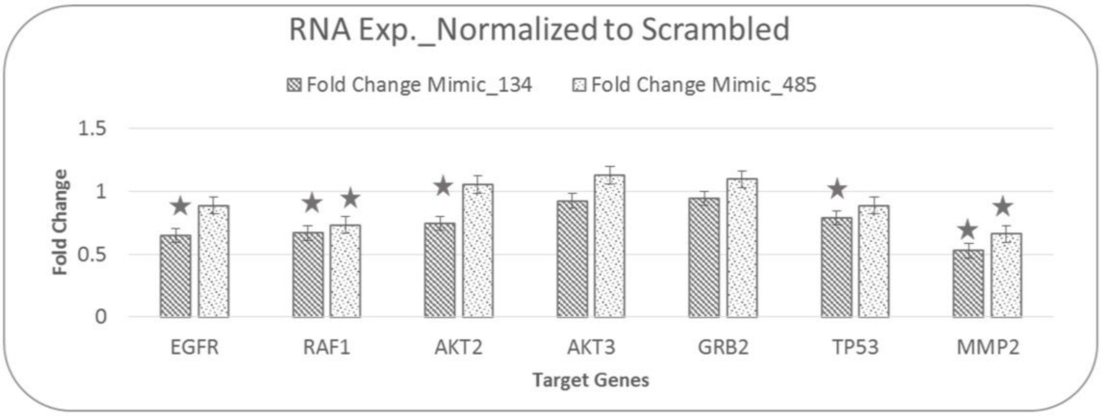


Real Time PCR and Western blot for key miRNA target genes. (PDF 94 kb). Target gene expression (Fold change compared to scrambled transfection) upon up-regulation of miR-134 and miR-485-5p via mimics in glioma cell line, at RNA and protein level. Error bars are indicated as standard error. The asterix marks denote downregulation of targets to 20% or more (Fold Change <= 0.8).

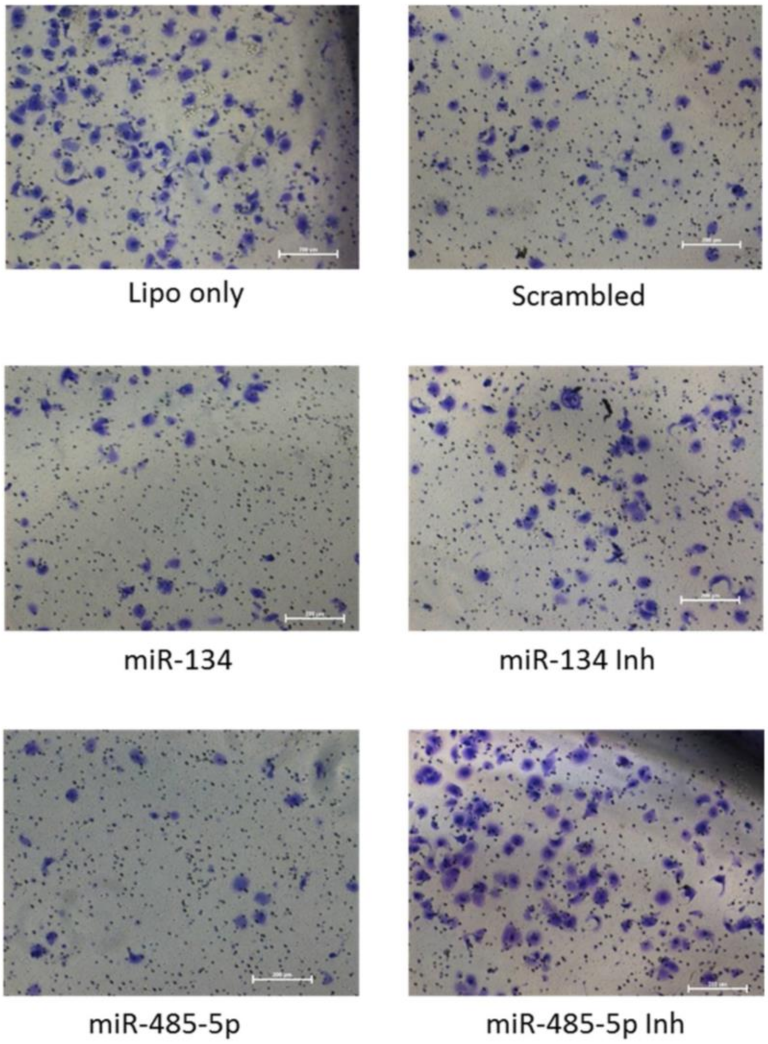


Representative image of Matrigel assay showing decreased invasion upon administration of miR-134 and miR-485-5p in glioma cells, as compared to scrambled. This phenotype is reversed upon addition of inhibiters of both the miRNAs. Scale is 200µm.

## Scrambled

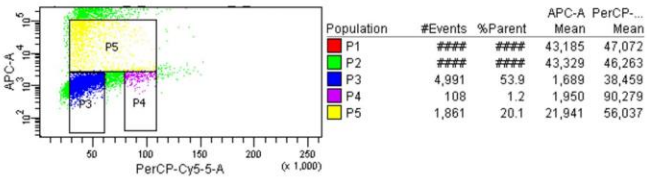


| Population | #Events | %Parent | APC-A Mean | PerCP-... Mean |
|---|---|---|---|---|
| P1 | #### | #### | 43,185 | 47,072 |
| P2 | #### | #### | 43,329 | 46,263 |
| P3 | 4,991 | 53.9 | 1,689 | 38,459 |
| P4 | 108 | 1.2 | 1,950 | 90,279 |
| P5 | 1,861 | 20.1 | 21,941 | 56,037 |

## miR-134

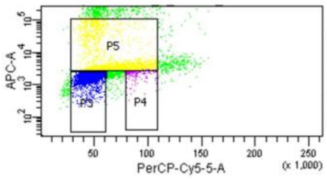


| Population | #Events | %Parent | APC-A Mean | PerCP-... Mean |
|---|---|---|---|---|
| P1 | #### | #### | 36,398 | 62,513 |
| P2 | #### | #### | 35,262 | 58,802 |
| P3 | 4,711 | 53.2 | 1,688 | 47,308 |
| P4 | 235 | 2.7 | 2,152 | 89,766 |
| P5 | 1,811 | 20.4 | 18,849 | 67,131 |

## miR-485-5p

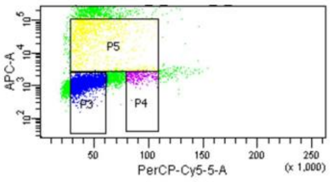


| Population | #Events | %Parent | APC-A Mean | PerCP-... Mean |
|---|---|---|---|---|
| P1 | #### | #### | 11,675 | 53,415 |
| P2 | #### | #### | 11,831 | 52,759 |
| P3 | 3,623 | 38.6 | 795 | 36,039 |
| P4 | 1,396 | 14.9 | 1,371 | 72,841 |
| P5 | 888 | 9.5 | 78,936 | 56,441 |

## miR-134 Inh.

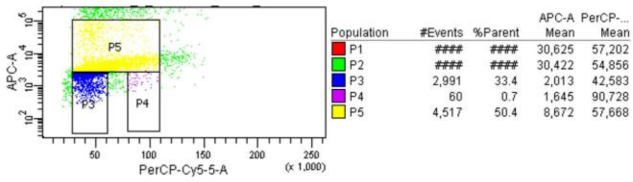


| Population | #Events | %Parent | APC-A Mean | PerCP-... Mean |
|---|---|---|---|---|
| P1 | #### | #### | 30,625 | 57,202 |
| P2 | #### | #### | 30,422 | 54,856 |
| P3 | 2,991 | 33.4 | 2,013 | 42,583 |
| P4 | 60 | 0.7 | 1,645 | 90,728 |
| P5 | 4,517 | 50.4 | 8,672 | 57,668 |

## miR-485-5p Inh.

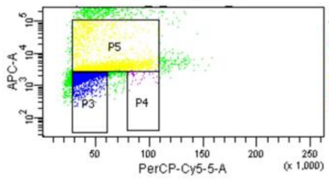


| Population | #Events | %Parent | APC-A Mean | PerCP-... Mean |
|---|---|---|---|---|
| P1 | #### | #### | 26,674 | 56,154 |
| P2 | #### | #### | 27,051 | 54,855 |
| P3 | 5,258 | 57.3 | 1,816 | 44,925 |
| P4 | 134 | 1.5 | 2,057 | 89,385 |
| P5 | 2,128 | 23.2 | 13,766 | 65,318 |

Representative image of BrdU assay showing decreased proliferation (Yellow; S phase) upon administration of miR-134 and miR-485-5p in glioma cells, as compared to scrambled. This phenotype is reversed upon addition of inhibiters of both the miRNAs.

**Scrambled**

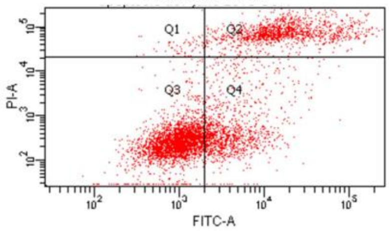


| Population | #Events | %Parent | FITC-A Mean | PI-A Mean |
|---|---|---|---|---|
| All Events | 10,000 | #### | 10,397 | 23,210 |
| Q1 | 83 | 0.8 | 1,251 | 41,610 |
| Q2 | 2,513 | 25.1 | 32,332 | 88,444 |
| Q3 | 4,495 | 45.0 | 1,012 | 447 |
| Q4 | 2,909 | 29.1 | 6,209 | 1,505 |
| P1 | 5,741 | 57.4 | 8,837 | 19,489 |

**miR-134**

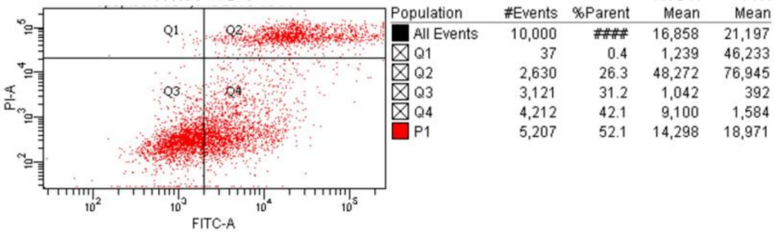


| Population | #Events | %Parent | FITC-A Mean | PI-A Mean |
|---|---|---|---|---|
| All Events | 10,000 | #### | 16,858 | 21,197 |
| Q1 | 37 | 0.4 | 1,239 | 46,233 |
| Q2 | 2,630 | 26.3 | 48,272 | 76,945 |
| Q3 | 3,121 | 31.2 | 1,042 | 392 |
| Q4 | 4,212 | 42.1 | 9,100 | 1,584 |
| P1 | 5,207 | 52.1 | 14,298 | 18,971 |

**miR-485-5p**

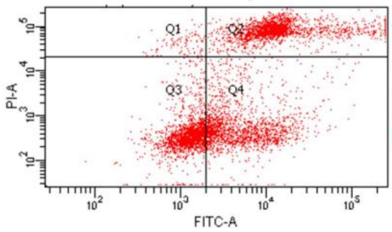


| Population | #Events | %Parent | FITC-A Mean | PI-A Mean |
|---|---|---|---|---|
| All Events | 10,000 | #### | 16,337 | 39,863 |
| Q1 | 129 | 1.3 | 1,145 | 46,274 |
| Q2 | 3,915 | 39.2 | 33,827 | 98,469 |
| Q3 | 2,724 | 27.2 | 1,141 | 688 |
| Q4 | 3,232 | 32.3 | 8,565 | 1,633 |
| P1 | 6,074 | 60.7 | 12,784 | 30,804 |

**miR-134 Inh**

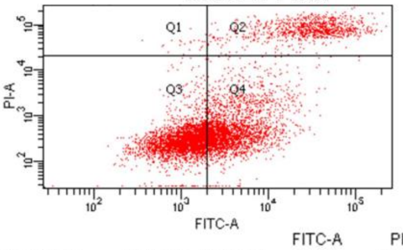


| Population | #Events | %Parent | FITC-A Mean | PI-A Mean |
|---|---|---|---|---|
| All Events | 10,000 | #### | 11,576 | 17,822 |
| Q1 | 29 | 0.3 | 1,237 | 39,010 |
| Q2 | 1,796 | 18.0 | 47,476 | 95,142 |
| Q3 | 3,982 | 39.8 | 1,065 | 376 |
| Q4 | 4,193 | 41.9 | 6,252 | 1,124 |
| P1 | 7,043 | 70.4 | 8,604 | 12,080 |

**miR-485-5p Inh**

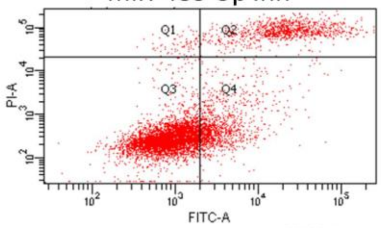


| Population | #Events | %Parent | FITC-A Mean | PI-A Mean |
|---|---|---|---|---|
| All Events | 10,000 | #### | 7,874 | 16,914 |
| Q1 | 113 | 1.1 | 1,296 | 43,731 |
| Q2 | 1,651 | 16.5 | 36,059 | 95,905 |
| Q3 | 5,950 | 59.5 | 876 | 409 |
| Q4 | 2,286 | 22.9 | 6,058 | 1,499 |
| P1 | 6,667 | 66.7 | 6,673 | 13,916 |

Representative image of AnnexinV-PI assay showing increased apoptosis upon administration of miR-134 and miR-485-5p in glioma cells, as compared to scrambled. This phenotype is reversed upon addition of inhibiters of both the miRNAs.

**Methodology**

**RNA Isolation and TLDA Assay**

In brief, the isolation protocol used organic extraction followed by purification on a silicate matrix under specialized binding and washing conditions, enabling isolation of small RNAs along with larger RNA species.

Each array of TLDA has MGB (minor groove binder) -labelled probes specific for all the miRs tested in the panel. The array was procured from Applied Biosystems Inc. snU6 was used as an internal control for relative quantification. Briefly, reverse transcription was done using 600ng of total RNA using multiplex primer pool for 47 mature miRNAs (Life Technologies), Multiscribe RT, RNase inhibitor and 100 nM dNTP. The reaction mixture was run for 30 min at 16°C, 30 min at 42°C and 5 min at 85°C.

Quantitative PCR was conducted in a 10µL volume containing 1/10 of the reversed transcribed product, 1x TaqMan Universal PCR master mix, and 1x TaqMan miRNA assay reagent. Amplification was performed at 95°C for 10 minutes, followed by 40 cycles of 95°C for 15 s, and 60°C for 1 minute on 7900HT Fast Real-Time PCR System (Applied Biosystem). Pre-processing of raw TLDA data files involved of threshold and baseline corrections for each sample, where each amplification plot was evaluated to confirm that the threshold cycle (Ct) value matched with the midpoint of logarithmic amplification (SDS 2.3, Life Technologies, Melbourne).

For Real time PCR after CRISPR-mediated modulation of C14-IP-3, SYBR green (KAPA biosystems) based assay was done. cDNA was prepared from 400ng total RNA isolated via MiRVana kit (Ambion), in a 10ul reaction volume, using QuantimiR kit (System Biosciences). Eight random miRs were taken from the cluster to assess the CRISPR modulation. Six miRs (four miRs from other genomic region, and two from C14MC but upstream of C14-IP-2) were selected upstream of the local regulator to assess specificity of the sgRNA targeting. Forward primers were designed for the miRNAs to be tested, and universal reverse primer was supplied with the QuantimiR kit. U6 snRNA was used as internal control for quantification of the miRNAs. The cocktails used were as follows: Cocktail 1: sg3 + sg8 + sg10, and Cocktail 2: sg 1 + sg2 + sg3.

**Contig building and genome assembly from ENCODE data**

Data for contig building was taken from polyA+ RNA sequencing of HUVEC cell line of ENCODE (GSM758565). Trimming and quality filtering of the raw files in FASTQ format was performed using FASTX tool kit (http://hannonlab.cshl.edu/fastx_toolkit/download.html) and FASTQC (http://www.bioinformatics.babraham.ac.uk/projects/download.html#fastqc). The sequenced reads were first mapped to the genome (hg19) using bowtie v0.12.8 using only non-junction reads to estimate the mean insert size of the library. Insert size is the stretch of the fragment which remains unread by the pair-end reads and it is the pre-requisite for further alignment on

the transcriptome. Reads were further mapped to reference transcriptome (NCBI build 37.1) using Tophat v2.0.5 with a maximum of two mismatches. In order to assemble contigs and their expression in terms of FPKM, genome assembly was performed using Cufflinks v2.0.2. These assembly and alignment files were further uploaded on IGV v2.2.10 to look for the possible contigs around the region of interest.

**Cloning into pgl3-Basic and pgl3-Promoter vectors**

Primers for the selected region were used to amplify the same from isolated genomic DNA (lab control). NheI and HindIII sites were introduced in the forward and reverse primers, respectively, to be used for pgl3-Basic, whereas NheI and XhoI sites were used when cloning into pgl3-Promoter vector. The PCR products were double digested for 3.5 hours at 37°C with the respective enzymes (sequential digestion for pgl3-promoter; with XhoI first at 37°C for overnight, followed by NheI for 3.5 hours at 37°C) and ligated into both the vectors (at 16°C for 16 hours). Pgl3-Basic has a firefly gene but no promoter, hence would be used to check promoter activity, while pgl3-Promoter has a promoter for the firefly gene, and any additive luminescence would suggest enhancer activity. These vectors were transformed by heat shock into DH5α strains and incubated at 37°C for overnight, followed by positive clone selection and confirmation by all three approaches (PCR using vector specific primers, restriction digestion and Sanger sequencing), post plasmid isolation.

**Cell Line Conditions**

U87-MG was maintained in MEM (SIGMA) supplemented with 10% FBS and 1% L-Glutamine, whereas LN229 was maintained in DMEM Glutamax High glucose (GIBCO) supplemented with 10% FBS, and cells were seeded for overnight, and each condition was put in duplicates. Transfection was done in Opti-MEM media. All cell culture materials were taken from GIBCO, unless otherwise mentioned.

**sgRNA generation, purification and transfection details**

Upon designing of the sgRNAs, they were procured from SIGMA. Anneal-extension PCR was done for generation of sgRNA template. 2.5µM of both forward and reverse primers, 1x PCR buffer, 5mM dNTPs, 0.3U Taq Pol, and nuclease free water to make the final reaction volume to 50µL were subjected to PCR. PCR conditions were as follows: denaturation at 95°C, for 3 mins, 30 rounds of PCR cycling at 95°C (10sec), 45°C (30sec), 72°C (30sec), final extension at 72°C for 7 mins and cooling at 4°C. Once the template was ready, the product was checked on agarose gel to verify the size, and proceed for *in vitro* transcription (IVT) of individual sgRNAs.

For IVT reaction, Invitrogen Ambion MEGAscript T7 transcription kit (Invitrogen) was used, where, 1x Reaction buffer, 0.133 mM each of UTP, ATP, GTP and CTP, 2µL of DMSO, 5µL

template of sgRNA template, 2µL of enzyme mix, and nuclease free water to make the final reaction volume to 20µL was mixed. The reaction mix was incubated at 37°C for overnight, or 5-6hours. After this step, the mix was subjected to another 30min incubation with 2µL Turbo DNAse at 37°C. Upon completion of this step, the mix was run on 2% agarose gel to check the sgRNA size and quality. This is followed by the final step of sgRNA purification.

Purification was done by NucAway spin columns (Thermo Fischer Scientific). For purification, 650µL water was put in the columns, and vortexed or tapped until all the bubbles were removed. The columns are kept at room temperature for 15mins, followed by spinning at 800g for 2mins. The columns were then transferred into a new 1.5ml vial and individual sgRNA was transferred into each column and kept for 5mins. The vials were then spinned down at 800g for 2mins, and 2µL of Superase-In (RNase inhibitor) was added. It was stored at -20°C until use.

For transfection, LN229 cells were seeded in six well plates, and kept overnight at their maintain conditions. Next day, 2ug of plamid (dCas9-VP64) was transfected using Lipofectamine 3000 (Invitrogen), as per manufacturer's recommendation, in a ratio of 1:1, in Opti-MEM media, and media was changed after 5hours. After 24hours of plasmid transfection, 2ug of sgRNA cocktail was transfected using Lipofectamine 3000 under similar conditions. The cells were harvested after 24hours of sgRNA transfection, for downstream processing.

**miRNA and mRNA interaction**

3' UTR clones of target genes (500ng) were procured from GeneCopoeia (USA), and miRNA mimics (30nM), scrambled (30nM) and miRNA inhibitors (60nM) from Dharmacon (GE-Dharmacon Inc., USA) were used for transfection, with 1:3 ratio of lipofectamine 3000 (Invitrogen Inc.) to DNA. The luciferase assay was done as explained above, except for the conditions tested, which were, MES (mimic + empty vector + scrambled), MTS (mimic + target gene + scrambled) and MTI (mimic + target gene + inhibitor).

Relative expression of the target genes were checked using qRT-PCR, upon individual overexpression of both microRNAs. cDNA was synthesized from 1µg total RNA using High Capacity cDNA Reverse Transcription Kit (Applied Biosystems), as per the manufacturer's protocol in a reaction volume of 20µl. For qPCR, 10x dilutions of the cDNA were used for each qPCR reaction. Primers for the target genes tested were taken from qPrimerDepot. Primer list given in Supplemental File. Relative quantitation was performed as before. The mean expression level of B2M was used for normalization. The differences in expression between miR transfection and scrambled transfection were calculated using the $2^{\Delta\Delta CT}$ method.

Total protein was isolated and western blot was performed. Total protein was isolated from the cell pellet after 48 hours of transfection of the miRNAs, using a mixture of RIPA buffer (SIGMA),

0.1M DTT and protease inhibitor, followed by incubation on ice (30 min, with constant agitation) and centrifugation at 4°C for 45 minutes. The supernatant containing the protein was collected. The total protein was quantitated using Qubit (Invitrogen), using manufacturer's protocol. Sample preparation was done prior to loading onto the gel, by denaturing the protein, by first adding the samples to buffer solution (4:1 ratio), mixed properly and subjecting them to 95°C for 5mins. The protein (40μg) collected from all the transfected conditions was resolved using a 10% SDS-PAGE at 80V for 1.5-2 hours. Wet transfer by methanol activated PVDF membrane (MDI) was done at 4°C (time depended on the size of the protein under study), followed by blocking the membrane with 5%BSA for 1 hour at room temperature. This was followed by primary antibody incubation (diluted in 5%BSA, according to the manufacturer's recommendation) at 4°C for overnight. The primary antibodies that were used were panAKT, AKT2, pAKT, EGFR, TP53, FAK, MMP9, GAPDH and β-actin. Three washes of the membranes was carried out using TBST and rocking them for 15mins each time, at room temperature. Secondary antibody incubation was done at recommended dilution as per the manufacturer, diluted in the blocking buffer, and incubated at room temperature for 1 hour. Three TBST washed was followed as explained earlier. Chromogenic substrate (DAB solution) was used for staining the membranes, and visualize the protein, followed by stopping the reaction by rinsing with water and treatment with 5% glacial acetic acid to ensure the longevity of blot. The quantitative analysis was done using ImageJ software. The details of the antibodies used have been given below.

**Survival Analysis**

Level 3 miRNA expression data and corresponding clinical information for GBM samples were taken from TCGA. Hazard curves for each miRNA were calculated using Kaplan-Meier survival function estimation method as implemented in Python package Lifelines. For calculation of survival function the samples were divided into two groups based on expression of the given miRNA. The partitioning were done such that samples with expression values above 80th percentile were placed in one group and those below 20th percentile were placed in the other group. The significance of difference between survival functions of the two groups was assessed using log rank test. The samples with survival more than 730 days were censored. Plotting of hazard curves and their 95% confidence interval was done using custom Python scripts. Principal component analysis was performed using Python's Scipy package.

| Details of Key miRNAs target gene primers used in Real Time PCR | | |
|---|---|---|
| **GeneName (mRNA-ID)** | **Primer Name** | **Sequence** |
| EGFR (NM_005228) | EGFR_RT_FP | GGGCTCTGGAGGAAAAGAAA |
| | EGFR_RT_RP | AAATTCCCAAGGACCACCTC |
| | | |
| RAF1 (NM_002880) | RAF1_RT_FP | GAACGACAGGACGTTGGG |
| | RAF1_RT_RP | GCTGATCGTCTTCCAAGCTC |
| | | |
| AKT2 (NM_001626) | AKT2_RT_FP | ACACAAGGAAAGGGAACCAG |
| | AKT2_RT_RP | ACCTAGCTCGGGACAGCTC |
| | | |
| AKT3 (NM_181690 & NM_005465) | AKT3_RT_FP | GCTCAGAGGGGAGTCATCAT |
| | AKT3_RT_RP | GGAAGTATCTTGGCCTCCAG |
| | | |
| GRB2 (NM_002086, NM_203506) | GRB2_RT_FP | CTACTGCAGACGACGAGCTG |
| | GRB2_RT_RP | AGTTCTTGGGAATGAAGCCG |
| | | |
| TP53 (NM_000546) | TP53_RT_FP | GAGCGTGCTTTCCACGAC |
| | TP53_RT_RP | TGTTTCCTGACTCAGAGGGG |
| | | |
| MMP2 (NM_004530) | MMP2_RT_FP | ATGCCGCCTTTAACTGGAG |
| | MMP2_RT_RP | GGAAAGCCAGGATCCATTTT |

| Details of antibodies used in western blot | | | |
|---|---|---|---|
| **ANTIBODY** | **SOURCE** | **CATALOGUE NO.** | **DILUTION** |
| AKT | M/S Cell Signaling | C67E7 | 1:500 |
| p-AKT | M/S Cell Signaling | S473 | 1:1000 |
| EGFR | M/S Cell Signaling | D38B1 | 1:1000 |
| FAK | M/S Cell Signaling | Y397 | 1:1000 |
| TP53 | M/S SantaCruz | SC126 | 1:250 |
| GAPDH | M/S SantaCruz | SC32233 | 1:1000 |
| β-ACTIN | M/S Sigma | A5441 | 1:1000 |
